# SOX2OT variant 7 contributes to the synergistic interaction between EGCG and Doxorubicin to kill osteosarcoma via autophagy and stemness inhibition

**DOI:** 10.1186/s13046-018-0689-3

**Published:** 2018-02-23

**Authors:** Wanchun Wang, Ding Chen, Kewei Zhu

**Affiliations:** Department of orthopedics, the second Xiangya hospital of Central South University, Remin road No.139, Changsha, Hunan China

**Keywords:** Osteosarcoma, Doxorubicin, EGCG, SOX2OT, Autophagy, Cancer stem cells, Notch3

## Abstract

**Background:**

Doxorubicin is the preferred chemotherapeuticdrug for osteosarcoma treatment of which clinical efficacy is limited because of its chemo-resistance and cardiac toxicity. It is necessary to develop the combination regimen with complementary molecular mechanisms to reduce the side effects and enhance sensitivity of Doxorubicin. EGCG is a polyphenol in green tea with antitumor bioactivity,which has been found that its combination with certain chemotherapeutic drugs could improve the antitumor efficiency.

**Methods:**

In this study, MTT assay was used to detect the cell growth inhibition The CD133+/CD44+ cells were isolated from U2OS and SaoS2 cell lines using magnetic-activated cell sorting and identified by flow cytometry analysis. qRT-PCR was used for determining the relative mRNA levels of key genes. Immunofluorescence was performed to evaluate the autophagy flux alterations. Self-renewal ability was accessed by sphere-forming assay. Tumorigenicity in nude mice was preformed to evaluate tumorigenicity in vivo.

**Results:**

We found that EGCG targeting LncRNA SOX2OT variant 7 produced synergistic effects with Doxorubicin on osteosarcoma cell growth inhibition. On the one hand, EGCG could reduce the Doxorubicin-induced pro-survival autophagy through decreasing SOX2OT variant 7 to improve the growth inhibition of Doxorubicin. On the other hand, EGCG could partially inactivate Notch3/DLL3 signaling cascade targeting SOX2OT variant 7 to reduce the stemness then abated drug-resistance of osteosarcoma cells.

**Conclusions:**

This study will help to reveal the molecular mechanisms of synergistic effects of EGCG and Doxorubicin on OS chemotherapy and improve the clinical efficacy of chemotherapy as well as provide a basis for developing antitumor drugs targeting osteosarcoma stem cells.

## Background

Osteosarcoma is the most common histological form of primary bone tumors in children and adolescents which originates from the malignant transformation of mesenchymal cells with high mortality [1]. It often occurs during the differentiation of osteoid tissue and immature osteoblast. Doxorubicin (Dox) is one of the most commonly used chemotherapeutic drugs for osteosarcoma [2]. However, the intrinsic weakness of DOX severely limits its clinical efficacy: low-dose usage could not only reduce its effectiveness but also lead to drug resistance, while dose increasement would cause severe cardiotoxicity [3]. Therefore, the overall survival rate of osteosarcoma patients is only 5 to 20% which is not satisfactory [4]. It is now generally recognized that the cancer stem cells (CSCs) could be a major cause of chemo-resistance and tumor recurrence [5]. Therefore, developing the combination regimen with potential complementary mechanisms, especially targeting CSCs, may be a promising avenue of drug toxicity reduction and efficacy improvement.Epigallocatechin gallate (EGCG) is the highest content of catechin in green tea with many physiological and pharmacological activities. It was found that EGCG could promote the sensitivity of traditional anticancer drugs [3, 6, 7] and reverse multidrug resistance [8]. Similarly, EGCG was reported to exert significant inhibitory effect on osteosarcoma cells including induce apoptosis, inhibit the proliferation and invasion of osteosarcoma cells [9–11].

Long non-coding RNAs have been reported to play important roles in tumor progression. Human SOX2 over lapping transcript (SOX2OT) gene can generate 8 lncRNA transcript variants (variant 1–8) which are functionally assumed to be correlated with cellular differentiation and carcinogenesis [12]. These variants show diverse expression profiles in different cell or tissue types [12]. It is worth mentioning that the SOX2OT harbors pluripotency regulator SOX2 and could positively regulate SOX2 expression [13, 14]. But so far, the expression and function of lncRNA SOX2OT variants in osteosarcoma is still unclear.

Our preliminary experiment surprised to find that the combination of EGCG and Dox could produce synergistic effect on osteosarcoma cell growth inhibition. Moreover, EGCG treatment resulted in LncRNA SOX2OT variant7 downregulation in a concentration-dependent manner. Based on above descreption, this study investigated the underlying molecular mechanisms of the synergistic effect between Dox and EGCG targeting SOX2OT variant 7 and found that EGCG could decrease the Dox treatement-induced pro-survival autophagy partly through inhibiting SOX2OT variant 7 to improve the growth inhibition of Dox on osteosarcoma cells. On the other hand, EGCG could inactivate Notch3/DLL3 signaling targeting SOX2OT variant 7 to reduce the stemness of OS cells and then abated drug-resistance of osteosarcoma cells.

## Methods

### Tumor cell culture and tumor sphere-forming culture

The SaoS2 and U2OS osteosarcoma cell lines were purchased from the Cell Culture Center, Shanghai Institutes for Biological Sciences, the Chinese Academy of Sciences (Shanghai, People’s Republic of China). The cells were maintained in DMEM supplemented with 10% FBS, 25 mM hydroxyethyl piperazine ethanesulfonic acid buffer, 100 U/mL penicillin, and 100 μg/mL streptomycin in ahumidifed atmosphere of 5% carbon dioxide at 37 °C.

In order to form osteosarcoma spheres, 5 × 10^3^ cells /well suspended OS cells were cultured in low-adhesive six-well plates in serum-free DMEM-F12 (1:1) medium supplemented with 20 ng/ml fibroblast growth factor (FGF; R&D systems), 20 ng/ml epidermal growth factor (EGF; R&D systems), B27 (1:50) (Invitrogen), N2 (1:100) (R&D systems), and 10 ng/ml LIF (Millipore) for 10 days (that is sphere-forming culture medium). In addition, fresh DMEM-F12 (1:1) medium, EGF, and bFGF were added every other day. A single-cell suspension derived from primary spheres was obtained for the secondary sphere formation with the same method.

### Patients and tissue samples

10 pairs of OS tissue samples and their relative adjacent tissues were collected from the second Xiangya hospital from Dec, 2015 to March, 2016. Unfortunately, RNA degradation was found in 4 pairs of tissues which could not be used in subsequent experiments. Use of these samples for all experiments was approved by the Ethics Committee of the 2nd Xiangya Hospital of Central South University.

### Expression of SOX2OT variants in OS tissues and SOX2OT-V7 overexpressed lentivirus generation

SOX2OT transcript variants were amplified separately using RT-PCR according to previous literature reports. U-87 MG cell line was used as positive control [13, 15, 16]. The SOX2OT V7 sequence was synthesized by BGI (Beijing, China). The synthetic V7 DNA fragment and lentiviral vector PGMLV-6395 were enzyme digested with BamH I/EcoRI,Ligation was performed to construct V7 overexpressed lentivirus recombinant plasmid. The following lentivirus package was performed by Auragene biotech (Changsha, China).

### MTT assay

Cells were plated at a density of 5000 per well of a 96-well plate, 24 hafter plating, cells were treated as the indicated concentrations. 20 ml MTT with a concentration of 5 mg/mlwas added to each well for an additional 4 hours. The blue MTT formazan precipitate was then dissolved in 150 μL of dimethyl sulfoxide per well with incubation for 10 min in a rotary platform at 37 °C. Cell proliferation inhibition ratio was calculated according to the absorbance at a wavelength of 570 nm (A value) in each well by ELISA analyzer (MK3, Themo, USA). Cell proliferation inhibition ratio (%) = (A value of control group–A value of treated group)/A value of control group× 100%.

### Analysis of in vitro drug interaction

According to previous study [3], MTT assay was used to detect the absorbance at the wavelength of 570 nm as the OD value. The coefficient of drug interaction (CDI) was calculated as follows: CDI = AB/(A × B). According to the absorbance of each group, AB is the ratio of the combination groups to control group; A or B is the ratio of the single agent group to control group. Thus, CDI value< 1, = 1 or > 1 indicates that the drugs are synergistic, additive or antagonistic, respectively.

### Quantitative RT-PCR

Total RNA was extracted from cells using TRIzol Reagent (Invitrogen, Carlsbad, CA, USA) according to the manufacturer’s instructions, and then the RNA was reverse transcribed using the PrimeScript RT Master Mix Perfect Real Time kit (TaKaRa, Dalian, China) to obtain the cDNA. Using the cDNA as the template, real-time PCR assay was performed using the pairs of primers listed in Table 1. The 20 μL real-time PCR reaction included 0.5 μL of cDNA template, 0.25 μL of Forward/Reverse primers respectively, 10 μL of RNase-free dH_2_O, and 8 μL of 2.5× Real Master Mix (SYBR Green I). The reaction conditions included a pre-denaturation step at 94 °C for 10 s, and 40 cycles of 94 °C for 15 s and 58 °C for 60 s. After the reaction, the data were subjected to statistical analysis.

**Table 1:** Primer sequence list

Gene	Name	Sequence
Atg5	Sense	TAAGTTTGGCTTTGGTTG
	Antisense	TTCCCTTTCAGTTATCTCAT
Atg7	Sense	ATGCCTGGGCATCCAGTGAACTTC
	Antisense	CATCATTGCAGAAGTAGCAGCCA
Beclin 1	Sense	TGTGGAATGGAATGAAATCAA
	Antisense	CCCCCAGAACAGTACAACGGC
SOX2OT-V7	Sense	TCTGTTCAGTATTTGGAAGAAAG
	Antisense	GCTTGGACCCGCGTG
SOX-2	Sense	CCCTGTGGTTACCTTTTCCT
	Antisense	AGTGCTGGGACATGTGAAG
Oct-4	Sense	TTCAGCCAAACGACCATCT
	Antisense	GCTTTGCATATCTCCTGAAGA
CD44	Sense	GGAGCAGCACTTCAGGAGGTTAC
	Antisense	GGAATGTGTCTTGGTCTCTGGTAGC
Nanog	Sense	CTCTCCTCTTCCTTCCTCCAT
	Antisense	TTGCGACACTCTTCTCTGC
Notch1	Sense	CCCGCCAGAGTGGACAGGTCAGTA
	Antisense	TGTCGCAGTTGGAGCCCTCGTTA
Notch2	Sense	CCCACAATGGACAGGACA
	Antisense	GAGGCGAAGGCACAATCA
Notch3	Sense	TCTCAGACTGGTCCGAATCCAC
	Antisense	CCAAGATCTAAGAACTGACGAGCG
Jagged1	Sense	GACACCGTTCAACCTGACAGTATTA
	Antisense	GTCACAGGCATAGTGTCCAAAGA
Jagged2	Sense	TCGGGCAGGAACTGTGAGAAGGC
	Antisense	AATCACAGTAATAGCCGCCAATCAGGT
DLL1	Sense	AGGGGTGGAGAAGCATCTGAAA
	Antisense	AACCTGCTCGGTCTGAACTCG
DLL3	Sense	ACGCCTGGCCTGGCACCTT
	Antisense	CCCTCTAGGCATCGGCATTCACC
DLL4	Sense	ACAGTGAAAAGCCAGAGTGTCGG
	Antisense	TGAGCAGGGATGTCCAGGTAGG
Hes1	Sense	CAGAAAGTCATCAAAGCCTATT
	Antisense	TTCAGAGCATCCAAAATCAG
Hey1	Sense	AGAGGAATAATTGAGAAGCG
	Antisense	CAAACTCCGATAGTCCATAG
β-actin	Sense	AGGGGCCGGACTCGTCATACT
	Antisense	GGCGGCACCACCATGTACCCT

The sequence information of all primers used for qRT-PCR detection in this study was listed in the table

### Western blot

Cells were lysed in cell lysate, and then centrifuged at 12,000×g for 20 min at 4 °C. The supernatant was collected and denatured. Proteins were separated in 10% SDS-PAGE and blotted onto polyvinylidene difluoride membrane (PVDF). The PVDF membrane was treated with TBST containing 50 g/L skimmed milk at room temperature for 4 h, followed by incubation with the primary antibodies against the LC3B (1:3000, ab51520, Abcam), Atg5 (1:1000, ab108327, Abcam), Atg7 (1:100,000, ab524721,Abcam), Beclin1 (1 μg/ml, ab62557, Abcam), P62 (1:500, 18,470–1-AP, Proteintech, China), OCT-4 (1:1000, Proteintech), ABCG2 (1:50, ab24115, Abcam), c-Myc (1:10,000, ab32072, Abcam), Sox2 (1 μg/ml, ab97959, Abcam) and anti-β-actin (1:1000, Cell signaling) respectively, at 37 °C for 1 h. Membranes were rinsed and incubated for 1 h with the correspondent peroxidase-conjugated secondary antibodies. Chemiluminescent detection was performed with the ECL kit (Pierce Chemical, Rockford, IL, USA). The amount of the protein of interest, expressed as arbitrary densitometric units, was normalized to the densitometric units of ß-actin.

### Purifcation of CD133+/CD44+ cancer stem cells

CD133/CD44 immunomagnetic double screening were improved and performed according to the methods reported in previous literature [17]. A single-cell suspension of Saos-2 and U2OS cells was incubated with magnetic microbeads-conjugated with the mouse anti-human CD133 monoclonal antibody (Miltenyi Biotec, USA) for 30 min. After washing, the CD133+ cells were separated using the magnetic cell sorting system (autoMACS; Miltenyi Biotec, USA). The purified CD133+ cells were expanded for 14 days by culturing and then harvested as a single-cell suspension to be incubated with magnetic microbeads-conjugated mouse anti-human CD44 monoclonal antibody (Miltenyi Biotec, USA) for 30 min. After washing, the CD44+ cells were separated using the magnetic cell sorting system as described above. This two-step isolation enabled us to obtain sufficient number of CD133+/CD44+ CSCs for the following experiment.

To verify the purity of the isolated CD133+/CD44+ CSCs, cells were stained according to the supplied antibody protocols. Mouse anti-Human CD133/1 (Clone: AC133)-PE and mouse anti-human CD44 (Clone: DB105)-FITC (Miltenyi Biotec, USA) were used. Then flow cytometry analysis was performed using a FACSCalibur instrument (Becton Dickinson).

### Self-renewal capacity assay

To evaluate the colony-forming ability of different cells, self-renewal capacity assay was performed. We seeded OS cells or CSCs into 96-well plates at a density of 1 × 10^3^ cells / well using sphere-forming culture medium with TGF-beta (20 ng/ml) and the formation of colonies was quantifed at 14 days after inoculation. Colonies with over 50 cells were counted under an Olympus microscope.

### Autophagy detection

In this study, autophagy levels were detected using immunofluorescence staining and MDC staining beside autophagy marker detection. Cells were cultured on cover glasses coated with 0.1% gelatin in PBS in 6-well tissue culture plates with DMEM. The cells were incubated overnight then washed with PBS, and fixed in 4% paraformaldehyde for 15 min. They were then permeabilized in 0.2% Triton X-100 for 10 min prior to blocking in 6% bovine serum albumin (BSA) for 30 min. For immunofluorescence staining, the cells were incubated overnight with anti-LC3B (1:200, 12741S, Cell Signaling, USA) at 4 °C, followed by incubation with goat anti-rabbit IgG (H + L)-Cy3 (1:200, SA012, Auragene) for 1 h at room temperature in dark. Nuclei were counterstained with DAPI (10 μg/ml, C0065, Solarbio, China). Finally, cells were analyzed using a confocal fluorescence microscope (BX50; Olympus, Japan) and the percentage of cells with LC3 puncta was calculated by taking advantage of Image-Pro Plus 6.0. When autophagic vacuoles were labeled with monodansylcadaverine (MDC), cells were incubated with 0.05 mM MDC in RPMI1640 at 37 °C for 10 min. After incubation, cells were washed three times with PBS and immediately analyzed with a fluorescence microscopeequipped with a filter system (V-2A excitation filter:380/420 nm, barrier filter:450 nm). Images were captured and imported into Adobe Photoshop.

### Animal treatments

Animal experiments were performed in strict accordance with the Guide for the Care and Use of Laboratory Animals of the second Xiangya hospital of Central South University. The protocol was approved by the Committee on the Ethics of Animal Experiments of the second Xiangya hospital of Central South University. NOD/SCID mice at age of 3–5 weeks, male, were maintained in pathogen-free conditions at animal facility. The OSCs with or without SOX2OT V7 overexpression were resuspended in serum-free medium and mixed with Matrigel at the ratio of 1:1. NOD/SCID mice were randomly divided into 4 groups (*n* = 3 per group). 1 × 10^5^ indicated cells were inoculated subcutaneously into the inguinal folds of NOD/SCID mice. 7 days after inoculation, tumor formation was evaluated by palpation of injection sites., thenthe mice that developed palpable tumors were intraperitoneally injected EGCG (30 mg.kg-1) in combination with or without Notch-3 knockdown lentivirus (Lv-Notch3-) at the injection site every 3 days for 3 times. At the end of experiment (21 days), the mice were sacrificed under deep anesthesia with pentobarbital. The tumors were then dissected and captured.

### Statistical analysis

Results from at least three independent experiments were expressed as mean ± SD. Statistical analysis of data from two groups was compared by two-tail t-test. Data from multiple groups wasperformed by one-way ANOVA, followed by Tukey post test. Statistical significance was determined as *P* < 0.05.

## Results

### Autophagy inhibition contributes to the synergistic interaction between EGCG and Doxorubicin to kill the osteosarcoma cells

**Fig. 1:**
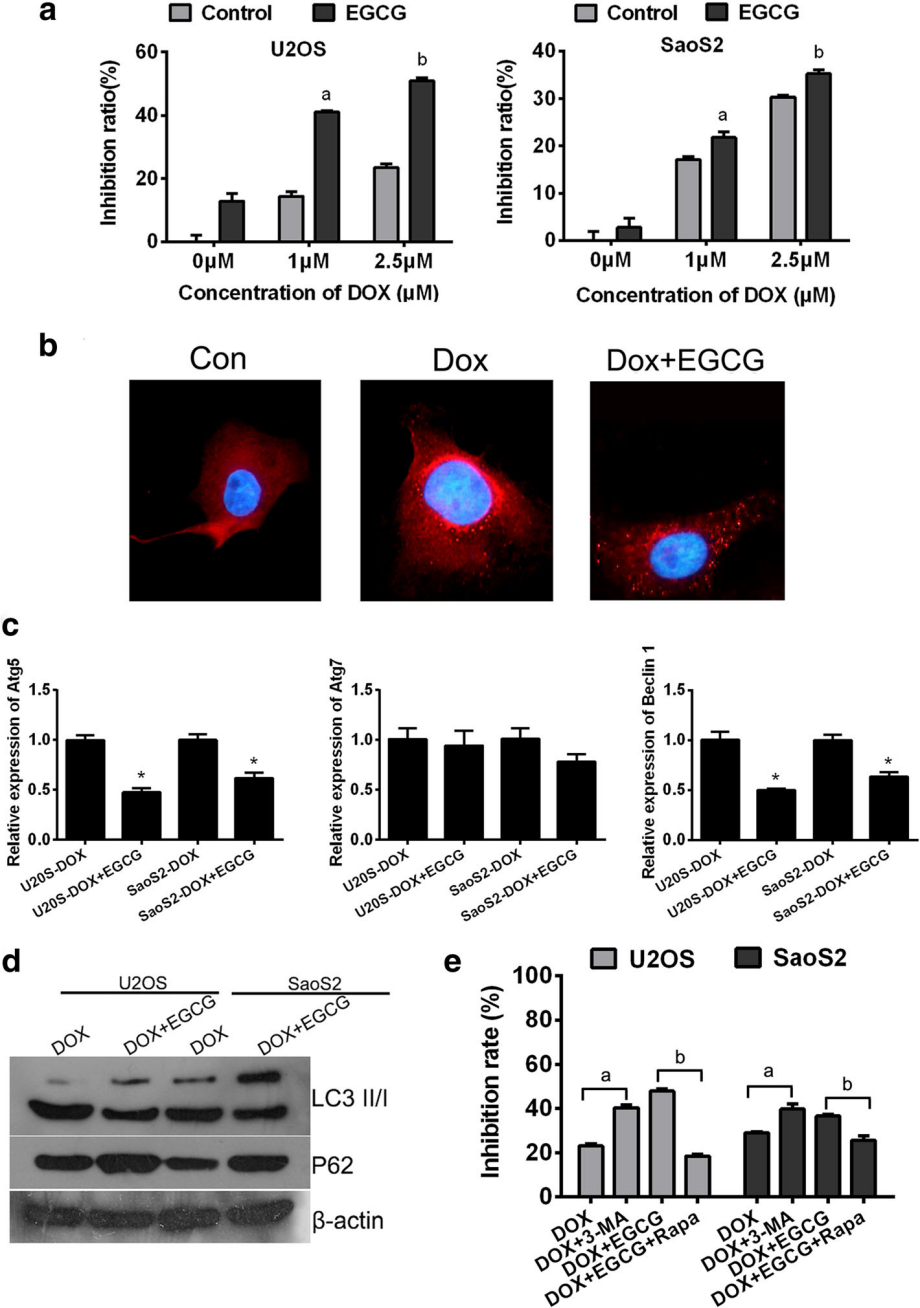
Autophagy inhibition contributes to synergistic interaction between EGCG and Doxorubicin to kill the osteosarcoma cells. **a** Combination of EGCG and DOX synergistically facilitate antitumor effects in U2OS and SaoS2 cell lines. Columns, percentage of inhibition ratio; bars, SE. Data was from a representative of 5 independent studies. ^a^ *p* < 0.05 vs. control group treated without EGCG but only 1 μm Dox, ^b^ *p* < 0.05 vs. control group treated without EGCG but only 2.5 μm Dox. **b** Immunofluorescence analysis indicated that elevated LC3 fluorescent punctual signals were visualized in cells administrated with 2.5 mM DOX, while when EGCG was added, the LC3 fluorescence signal becomes weaker. **c** Exposed to 2.5 μM Dox with or without 20 μg/ml EGCG for 24 h, the expression levels of Atg5, Atg7 and Beclin1 in OS cells were determined at the mRNA levels by qRT-PCR. Results shown are representative of three independent experiments and error bars indicate SE. ^*^ *p* < 0.05 vs. DOX single treated group. **d** Cell lysates following DOX treatment with or without EGCG were subjected to western blotting. Protein ratios normalized to β-actin were used to quantify fold change. **e** Cells were under different treatment, then MTT assay was performed to detect the growth inhibition. Data was from a representative of 5 independent studies. ^a^ *p* < 0.05 vs. DOX treated group without 3-MA, ^b^ *p* < 0.05 vs. combined treated group without Rapa

In order to determine whether EGCG could produce synergistic effects with Dox on osteosarcoma cell growth inhibition, MTT assays were applied and the coefficient of drug interaction (CDI) was calculated. The results showed either for U2OS or SaoS2 cell lines, when Dox was used in combination with EGCG, the growth inhibition was significantly up-regulated (Fig. 1a). As expected, according to the MTT assay and the coefficient of drug interaction (CDI, shown in Table 2), EGCG and DOX yielded synergistic interactions (CDI<1). As reported previously, autophagy contributed to the synergistic antitumor effects of EGCG and DOX [3], autophagy levels were detected in various ways. Firstly, immunofluorescence staining was performed to detect the LC3 translocation in U2OS cells. The results showed that in control group, LC3 was uniformly distributed in the cytoplasm, while LC3 expression occurred predominantly in punctuate dot-like structures when treated with Dox which wasconsistent with autophagy induction. Whereas the LC3 puncta decreased obviously in U2OS cells treated with EGCG combined with DOX (Fig. 1b). Secondly, the expression of autophagy markers were detected using qRT-PCR and western blot. It was found that, the relative mRNA levels of Atg5 and Beclin-1 were decreased significantly when OS cells were treated with Dox and EGCG together but there was no obvious expression alteration of Atg7 (Fig. 1c). The decrease in LC3II/I ratio and the simultaneous increase of autophagy substrate P62 suggested that autophagy levels were attenuated to some exent in the process of combining Dox with EGCG in both OS cell lines (Fig. 1d). In order to further define the synergistic growth inhibition between EGCG and DOX on OS cells was caused by autophagy mediation, cells were pre-treaed with 3-MA or rapamycin (Rapa) respectivly in different experimental groups. The MTT results showed that when pre-treated those cells with 3-MA, a representative autophagic antagonist, the DOX-induced proliferative inhibition effects was dramatically aggravated, while when pre-treated those cells with Rapa, a representative autophagic agonist, the synergistic growth inhibition effect of concurrent administration of EGCG andDox was significantly decresed (Fig. 1e). The experimental results illustrated that EGCG-induced autophagy inhibition contributes to the synergistic interaction between EGCG and Doxorubicin to kill the osteosarcoma cells.

**Table 2:** Dosage inhibitory effects of both EGCG and DOX on the cells (*n* = 5)

Drug	Concentration	Growth inhibitory effects (OD)	CDI
U2OS			
DOX	0 μM	0.876 ± 0.042	–
	1 μM	0.750 ± 0.027	–
	2.5 μM	0.670 ± 0.023	–
DOX + EGCG	0 μM + 20 μg/ml	0.763 ± 0.046	–
	1 μM + 20 μg/ml	0.515 ± 0.008	0.904 ± 0.053
	2.5 μM + 20 μg/ml	0.430 ± 0.019	0.855 ± 0.052
SaoS2			
DOX	0 μM	1.224 ± 0.050	–
	1 μM	1.014 ± 0.018	–
	2.5 μM	0.853 ± 0.012	–
DOX + EGCG	20 μg/ml	1.190 ± 0.054	–
	1 μM + 20 μg/ml	0.957 ± 0.032	0.794 ± 0.035
	2.5 μM + 20 μg/ml	0.793 ± 0.024	0.782 ± 0.036

Drug interaction was measured as described in materials and methods with increasing concentrations DOX or both agents for 48 h. CDI<1 indicates a synergistic effect, CDI = 1 indicates an additive effect, CDI>1 indicates an antagonistic effect

### Up-regulation of SOX2OT variant 7 in OS tumor tissues and cell lines

**Fig. 2:**
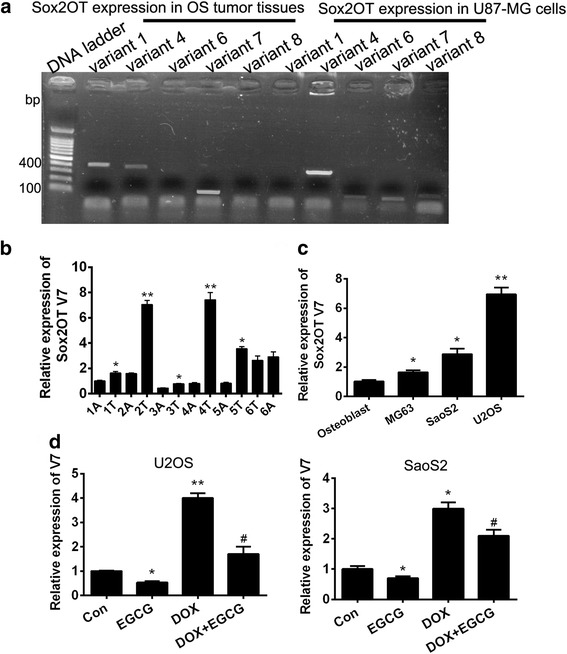
Up-regulation of SOX2OT variant 7 in OS tumor tissues and cell lines. Specific expression of SOX2OTs (variants 1, 4, 6, 7, and 8) in OS tumor tissues. **a** The expression of SOX2OTs in OS tumor tissue mixture was checked by qRT-PCR. The U-87 cell line was used as positive control. **b** qRT-PCR was performed to detect the SOX2OT V7 in 6 pairs of OS tissues (No.1–6), A means adjacent and T means timorous. Results shown are representative of three independent experiments and error bars indicate SE. ^*^ *p* < 0.05 vs. relative adjacent tissue ^**^ *p* < 0.01 vs. relative adjacent tissue. **c** qRT-PCR was performed to detect the mRNA level of SOX2OT V7 in 3 OS cell lines and primary osteoblast. Results shown are representative of three independent experiments and error bars indicate SE. ^*^ *p* < 0.05 vs. primary osteoblast group, ^**^ *p* < 0.01 vs. primary osteoblast group. **d** qRT-PCR was performed to detect the mRNA level of SOX2OT V7 in U2OS and SaoS2 cells with different treatment. Results shown are representative of three independent experiments and error bars indicate SE. ^*^ *p* < 0.05 vs. control group, ^**^ *p* < 0.01 vs. control group. ^#^ *p* < 0.05 vs. DOX single treated group

Using qRT-PCR, we initially examined the expression pattern of newly identified human SOX2OT variants (transcripts 1/4/6/7/8) in 6 OS tumor tissue mixture, with specific primers for each variant [13]. A glioblastoma multiform cell line U-87 was utilized as a positive control to compare the expression pattern of the variants.. As it is shown in Fig. 2a, no or very low expression was observed for SOX2OT transcripts 6 and 8. However, variant 1 showed a moderately high and variant 4 and 7 presented significantly high expression levels in OS tumor tissues. The expression pattern was different from that of U-87 which was used as a positive control.

The relative expression of the two most abundantly expressed SOX2OT variants (transcripts 1 and 7) were measured in OS tumor tissues and their responding adjacent tissues (*n* = 6) by qRT-PCR. The data was normalized to samples from adjacent tissues (A1) obtained from patient NO.1 (n = 6). The data revealed that SOX2OT variants 7 (V7) increased in 5/6 tumor tissues compared with their relative adjacent tissues (Fig. 2b). Besides, the relative mRNA levels of V7 were significantly in MG63, U2OS and SaoS2 cells when compared with primary osteoblast (Fig. 2c). These results indicated that V7 was overexpressed in osteosarcoma to some extent. It is noteworthy that the relative mRNA levels of V7 up-regulated obviously in OS cells treated with DOX but down-regulated in EGCG treated OS cells compared to control group. Meanwhile, the EGCG could decrease the V7 expression induced by DOX treatment significantly (Fig. 2d). These results suggested that V7 may be one of the important targets for EGCG to increase efficiency of DOX.

### EGCG inhibited DOX-induced autophagy by targeting SOX2OT V7 partially

**Fig. 3:**
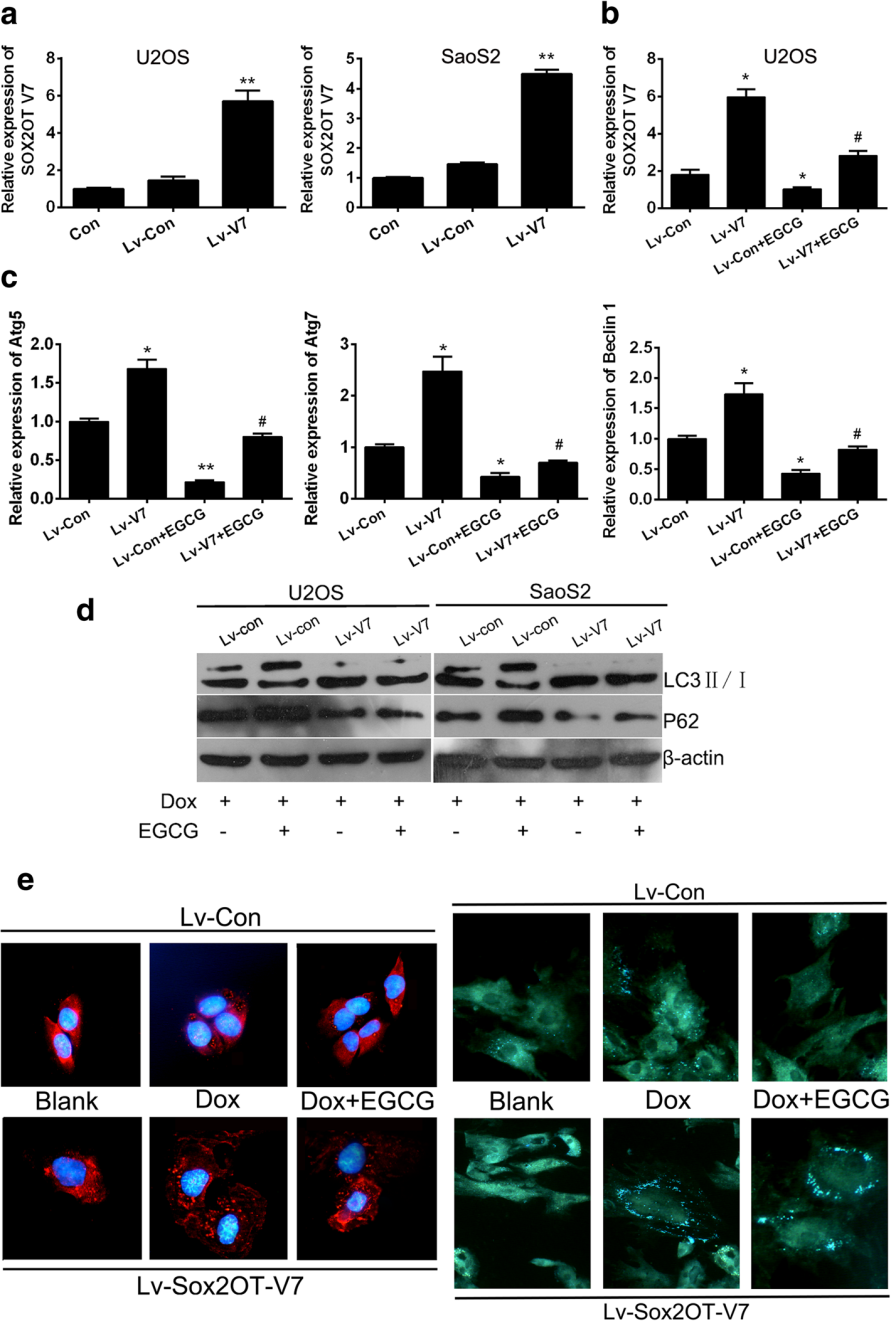
EGCG inhibited DOX-induced autophagy by targeting SOX2OT V7 partially. SOX2OT V7 gain-of-function cell models were constructed by lentivirus infection. **a** The relative mRNA levels of SOX2OT V7 in OS cells were checked by qRT-PCR. ^**^ *p* < 0.01 vs. Lv-Con. **b** qRT-PCR was performed to detect the SOX2OT V7 in U2OS cells treated with or without EGCG. Results shown are representative of three independent experiments and error bars indicate SE. ^*^ *p* < 0.05 vs. Lv-con, ^#^ *p* < 0.05 vs. Lv-con treated with EGCG. **c** qRT-PCR was performed to detect the mRNA level of autophagy associated genes in V7 over-expressed U2OS cells. Results shown are representative of three independent experiments and error bars indicate SE. ^*^ *p* < 0.05 vs. Lv-con, ^#^ *p* < 0.05 vs. Lv-con treated with EGCG. **d** Western blots were performed to detect the expression of LC3 and P62 in V7 over-expressed U2OS and SaoS2 cells with different treatment. **e** LC3 immunofluorescence (the left half) and MDC staining (theright half), LC3 puncta distribution and MDC signals were visualized by confocal fluorescence microscopy, and representative images are shown. The images were captured at 400 x magnification

In order to explore the biological function of SOX2OT V7 in osteosarcoma, V7 gain-of-function OS cell models were established by V7 overexpressed lentivirus infection. qRT-PCR was performed to verify the efficiency of V7 overexpression. The results showed that in V7 gain-of-function cell models, mRNA level of SOX2OT-V7 enhanced significantly in both cell lines (Fig. 3a). At the same time, it was found that EGCG treatment could significantly reduce the endogenous expression level of V7 in both parental and V7 overexpressed OS cells. Therefore, V7 is one of the targets of EGCG in osteosarcoma cells. To investigate whether V7 is an autophagy regulator in OS cells, relative mRNA levels of autophagy associated genes Atg5, Atg7 and Beclin1 were detected using qRT-PCR. The results demonstrated that the relative mRNA levels of Atg5, Atg7 and Beclin1 were all up-regulated significantly in SOX2OT V7 overexpressed U2OS cells compared with control cells regardless of EGCG treatment (Fig. 3c). In order to explore whether the autophagy regulation of V7 is associated with the synergistic effects of EGCG and DOX, western blots, immunofluorescence staining and MDC staining were performed in SOX2OT V7 overexpressed OS cells under DOX treatment with or without EGCG. The results showed that, when V7 was overexpressed in OS cells, the ratio of LC3II/I increased obviously and the autophagy substrate P62 decreased significantly which explained that SOX2OT V7 could induce autophagy in OS cells again. On the other hand, it was interesting that the inhibitory effect of EGCG on DOX-induced autophagy became faint in V7 overexpressed OS cells, the difference of the ratio of LC3II/I and the expression of P62 was not obvious between V7 overexpressed and control cells treated with DOX in combination with EGCG (Fig. 3d). Autophagy induction is associated with LC3, conjugated LC3 moves into autophagosomes and tightly binds to the autophagosome membrane. Thus, LC3 translocation is a reliable biomarker of autophagy [18]. Immunofluorescence staining was performed to detect the LC3 translocation. Meanwhile, MDC staining was another commonly used means of autophagy detection. The results showed that in control cells (Lv-Con), EGCG treatment could reduce the LC3 puncta and the MDC fluorescence signals obviously compared with DOX single treatment groups. While when V7 was overexpressed in OS cells, both the LC3 puncta and the MDC fluorescence signals enhanced obviously compared with the control group (Lv-Con). While, the difference of LC3 puncta and the MDC fluorescence signals were not obvious between V7 overexpressed (Lv-SOX2OT-V7) and control cells (Lv-Con) treated with DOX in combination with EGCG (Fig. 3e).The above results basically prove that EGCG inhibited DOX-induced autophagy by targeting SOX2OT V7 partially.

### EGCG reduced the expression of CSC markers of osteosarcoma stem cells

**Fig. 4:**
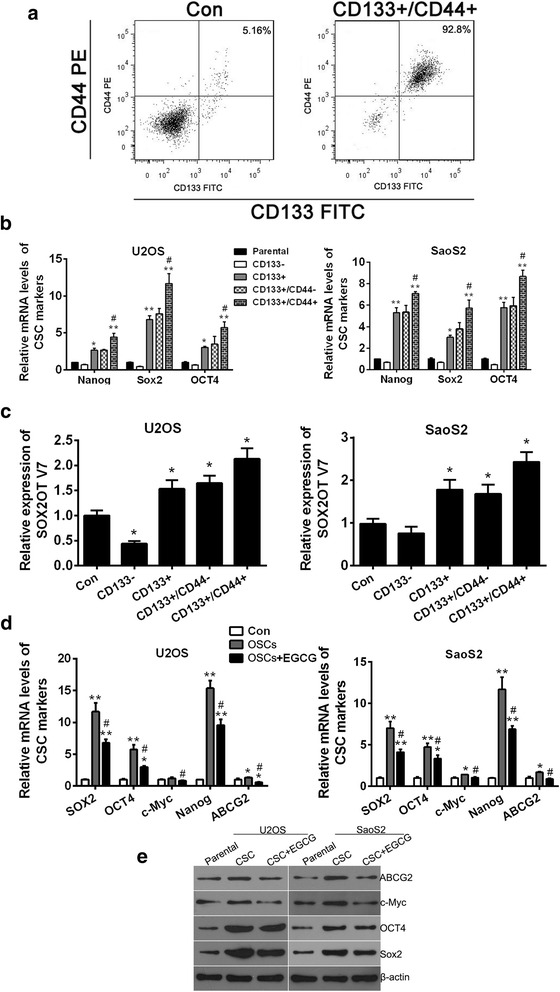
EGCG reduced the expression of CSC markers of osteosarcoma stem cell. **a** OSCs obtained from osteosarcoma cell line U2OS by CD133+/CD44+ immunomagnetic beads double positive screening and the isolated CD133+/CD44+ OSCs were identified by flow cytometry analysis. U2OS parental cells were set as Control (Con). **b** The relative mRNA levels of CSC markers Nanog, Sox2 and OCT4 in different bead-sorted cells were checked by qRT-PCR. ^*^ *p* < 0.05 vs. parental cells, ^**^ *p* < 0.01 vs. parental cells, ^#^ *p* < 0.05 vs. CD133+ cells. **c** qRT-PCR was performed to detect the SOX2OT V7 in different bead-sorted cells. ^*^ *p* < 0.05 vs. parental cells (Con). **d** Relative mRNA levels of CSC markers Nanog, Sox2, OCT4, c-Myc and ABCG2 in OSCs treated with or without EGCG were detected by qRT-PCR. ^*^ *p* < 0.05 vs. parental cells (Con), ^**^ *p* < 0.01 vs. parental cells (Con), ^#^ *p* < 0.05 vs. OSC. Results shown are representative of three independent experiments and error bars indicate SD. **e** Expression of CSC markers Nanog, Sox2, OCT4, c-Myc and ABCG2 in OSCs treated with or without EGCG were detected by western blots

Cancer stem cells (CSCs) is a small subset of cancer cells characterized by self-renewal capability and pluripotent differentiation potential. Therefore, CSCs are believed to be of great importance in carcinogenesis, metastasis, drug-resistance and cancer recurrence. The existence of CSCs in osteosarcoma that are capable of forming suspended spherical, clonal colonies, preferentially express CSC key marker genes has been demonstrated [19]. EGCG could inhibit SOX2OT V7 which is closely related to pluripotency regulator SOX2 of CSCs, therefore, we speculated that EGCG would exert certain inhibitory effect on osteosarcoma stem cells (OSCs). CD133 is considered to be the most common CSC marker, and has been used repeatedly for the successful isolation of osteosarcoma stem cells [19]. A growing number of studies have found that using immunomagnetic double positive screening could obtain CSCs with higher purity and stemness [20, 21]. In this study, stem cell like cells were obtained from osteosarcoma cells by CD133+/CD44+ immunomagnetic beads double positive screening method and the isolated CD133+/CD44+ CSCs were identified by flow cytometry analysis. The results showed that after immunomagnetic double positive screening, the percentage of CD133+/CD44+ positive cells reached over 90% in U2OS (Fig. 4a) parental cells, which was significantly higher than that before screening. In order to further verify the relative stemness of cells after immunomagnetic beads screening, qRT-PCR was used to detect the relative mRNA levels of conventional OSC markers. The results showed the mRNA levels of Nanog, OCT4 and Sox2 were all upregulated significantly after CD133+ screening compared with their parental cells. It is worth mentioning that, after further CD44+ screening of CD133+ cells, further significant upregulation of Nanog, OCT4 and Sox2 was observed in CD133+/CD44+ cell compared with CD133+ cells (Fig. 4b). The results also illustrated to some extent that CD133+/CD44+ double positive screening was more effective than CD133+ single screening. On the other hand, mRNA level of SOX2OT V7 was also detected in immunomagnetic bead-screened cells and was found with similar alteration to those of OSC markers.(Fig. 4c). Subsequently, the screened CD133+/CD44+ cells were termed as OSCs. To determine whether EGCG could inhibit osteosarcoma stem cells in vitro, qRT-PCR and western blot were performed to detect the expression of OSC markers. The results showed the Nanog, OCT4, Sox2, c-Myc and ABCG2 were all upregulated significantly in OSCs compared with parental cells (Con), but their expression reduced obviously after EGCG addition compared with OSCs without EGCG treatment (Fig. 4d and e).

### SOX2OT V7 is one of the important targets of EGCG inhibiting OSCs

**Fig. 5:**
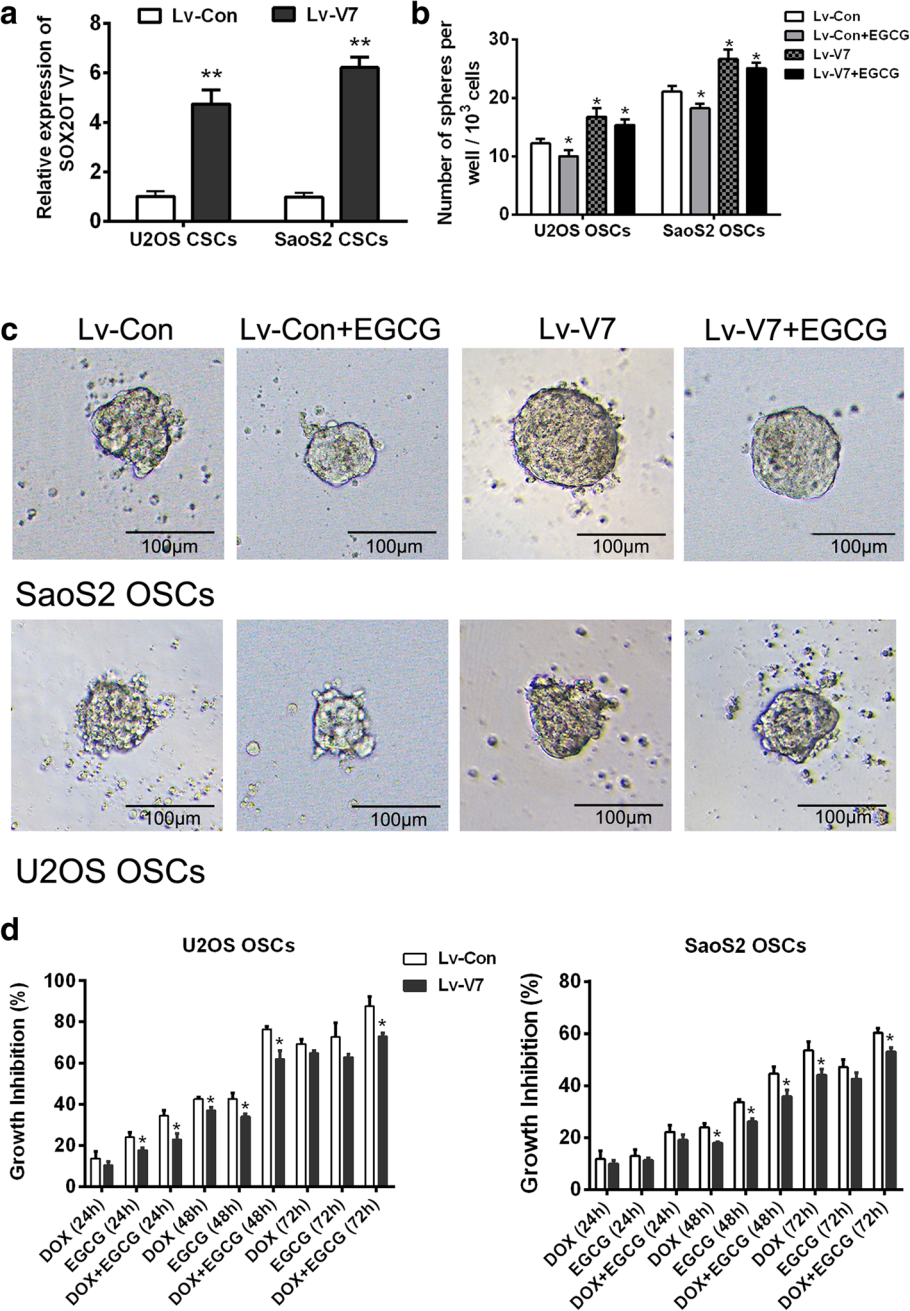
SOX2OT V7 is one of the important targets of EGCG inhibiting OSCs. OSCs screened from U2OS and SaoS2 were infected with V7 overexpression lentivirus (Lv-V7) to generate gain-of-function OSC models of SOX2OT V7. **a** qRT-PCR was performed to detect the mRNA level of SOX2OT V7 in V7 overexpressed OSCs. ^**^ *p* < 0.01 vs. control lentivirus infected OSCs (Lv-con). The sphere clone formation assay was performed to evaluate the self-renewal ability of OSCs. **b** 1 × 10^3^ OSCs were inoculated in 96-well plate and number of spheres per well was calculated on day 14. ^*^ *p* < 0.05 vs. control lentivirus infected OSCs (Lv-con). **c** Representative pictures of a tumor spheres were taken on the day14, under 100 × magnification, the bar = 100 μm. **d**. MTT assay was performed to study the inhibitory effect of EGCG and DOX, alone or in combination on OSC growth. ^*^ *p* < 0.05 vs Lv-con OSCs which were treated under the same treatment condition

After confirming that EGCG did inhibit OSCs, it was our next concern that the inhibition was partly targeting SOX2OT V7. OSCs screened from U2OS and SaoS2 were infected with V7 overexpressed lentivirus (Lv-V7) to generate gain-of-function OSC models of SOX2OT V7. qRT-PCR results showed that SOX2OT was successfully overexpressed in OSCs from both U2OS and SaoS2 (Fig. 5a). The sphere clone formation assay was performed to evaluate the self-renewal ability of OSCs. The results showed EGCG treatment could abate the tumor sphere formation ability of OSCs and V7 overexpression could enhance the spheroid formation ability in OSCs obviouly not only in sphere number but also in spheroid size. While, when the V7 overexpressed OSCs were treated by EGCG with the same condition, he alteration of sphere formation ability was no longer obvious compared with V7 overexpressed OSCs treated without EGCG (Fig. 5b and C). Then, MTT assay was performed to study the inhibitory effect of EGCG and DOX, alone or in combination on OSC growth. It was demonstrated that EGCG could inhibit the growth of OSCs which seems slightly stronger than that of DOX. V7 over-expression in OSCs could reduce the growth inhibitory effect of both DOX and EGCG when they were single used. At the same time, the OSC growth inhibition effect of EGCG combined with DOX was also significantly decreased after overexpression of V7 in OSCs (Fig. 5d). These results suggested that EGCG inhibited the stemness of OSCs, and SOX2OT V7 was one of the important targets for the OSC inhibition of EGCG.

### The OSC inhibitory effect of EGCG targeting SOX2OT V7 is partly achieved by restrain of Notch3 / DLL3 signaling

**Fig. 6:**
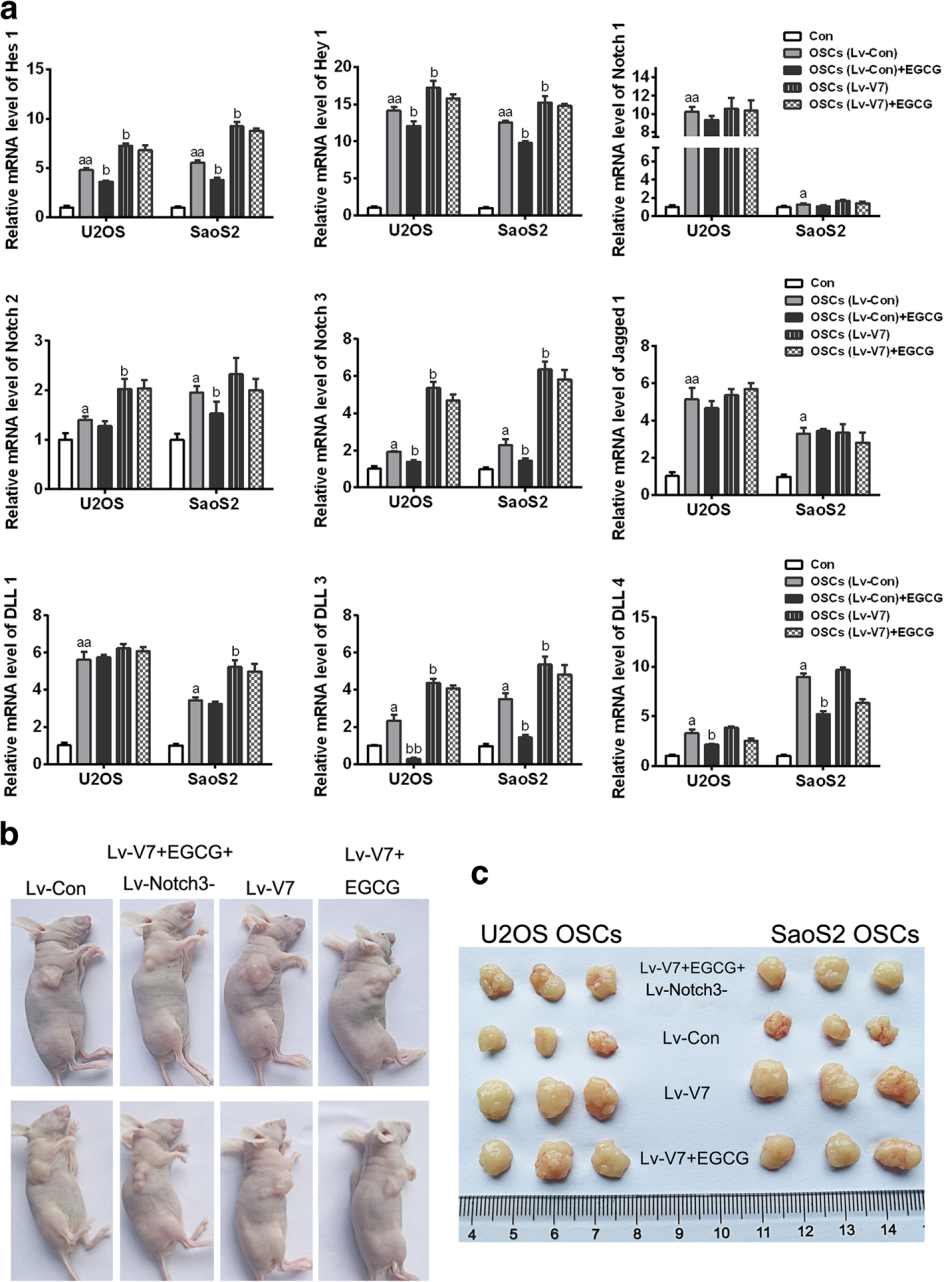
The OSC inhibitory effect of EGCG targeting SOX2OT V7 is partly achieved by restrain of Notch3 / DLL3 signaling. To illustrate whether the OSC inhibitory effect of EGCG targeting SOX2OT V7 is partly achieved by mediating of Notch signaling, relative mRNA levels of target genes, receptors and ligands of Notch signaling were detected using qRT-PCR in V7 overexpressed OSCs with or without EGCG treatment **a**.^a^ *p* < 0.05 vs. parental cells (Con), ^aa^ *p* < 0.01 vs. parental cells (Con), ^b^*p* < 0.05 vs. control lentivirus infected OSCs (Lv-con).**b** Typical pictures of nude mice with tumorigenicity which were under different treatments. **c** Typical pictures of stripped tumors derived from mice in different treatment groups

Notch signaling pathway plays an essential role in the development of cancer. Besides, Notch signaling pathway is also one of the classic signaling pathways of cancer stem cells. It was reported that EGCG could down-regulate the expression of Notch signaling genes and target genes [22–24]. To illustrate whether the OSC inhibitory effect of EGCG targeting SOX2OT V7 is partly achieved by mediating of Notch signaling, relative mRNA levels of target genes, receptors and ligands of Notch signaling were detected using qRT-PCR in V7 overexpressed OSCs with or without EGCG treatment. The results showed that the mRNA level of Notch target genes Hey1 and Hes1 enhanced significantly in OSCs compared with their parental cells. Besides, Hes1 and Hey1 expression further increased in V7 overexpressed OSCs compared with Lv-con OSCs. The results suggested V7 overexpression could further activated Notch signaling which has been active in OSCs. On the other hand, EGCG treatment could depresse the mRNA levels of Hes1 and Hey1 in OSC control groups which indicated that EGCG inhibited the Notch signaling pathway in OSCs. However, the inhibitory effects of EGCG on Hes1 and Hey1 were not significant in V7 overexpressed OSCs which showed that overexpression of V7 was associated with a significant reduction in the inhibitory effect of EGCG on Notch signaling. To further understand the factors involved in this process, Notch receptors (Notch1/2/3) and ligands (Jagged1/2, Dll1/3/4) were invested by qRT-PCR. The results showed only relative expression of Notch3 enhanced obviously compared with Lv-con group when V7 was overexpressed in OSCs. Meanwhile,EGCG treatment reduced Notch 3 expression in Lv-con group cells which was almost abolished when V7 was overexpressed in OSCs. In terms of ligand, only DLL3 showed similar expression changes with Notch3 (Fig. 6a). Based on the above experimental results, we speculated preliminarily that Notch3 / DLL3 signaling plays a role in OSC inhibitory effect of EGCG targeting SOX2OT V7.

Then we used nude mouse tumorigenicity assay to further verify the above results (Table 3). It was demonstrated that, when being inoculated the same number of OSCs, V7 overexpressed OSCs induced tumors with larger volume compared with Lv-con group cells. In the course of tumorigenesis, EGCG was injected into nude mice inoculated with Lv-V7 OSCs which did not reduce the tumor size obviously. It is worth noting that the tumor size was much smaller when EGCG was injected into nude mice inoculated with Lv-V7 OSCs in combination of Notch3 knockdown lentivirus (Lv-Notch3-). This result was proved again that OSC inhibitory effect of EGCG targeting SOX2OT V7 was partly achieved by restrain of Notch3 to some extent (Fig. 6b and C).

**Table 3:** Xenotransplantation of cells into NOD/SCID mice

OSC groups	Inoculum size	Tumor incidence at D7	Injection treatment From D7 to D21	Tumor incidence at D21	Tumor weight (g)
U2OS					
2003Lv-Con	1 × 10^5^	3/3	None	3/3	0.562 ± 0.046
Lv-V7	1 × 10^5^	3/3	None	3/3	1.386 ± 0.027
			EGCG	3/3	1.335 ± 0.031
			EGCG+Lv-Notch3-	3/3	0.844 ± 0.036
SaoS2					
Lv-Con	1 × 10^5^	3/3	None	3/3	0.499 ± 0.023
Lv-V7	1 × 10^5^	3/3	None	3/3	1.456 ± 0.034
			EGCG	3/3	1.360 ± 0.022
			EGCG+Lv-Notch3-	3/3	0.860 ± 0.056

## Disscussion

Osteosarcoma is the most common primary malignant bone tumor. One of the most active drugs for OS treatment is doxorubicin which is invariably included in OS chemotherapy protocols. Several studies have shown that high grade osteosarcoma patients may be inherently resistant to doxorubicin or become un-responsive to this drug during chemotherapy [25]. So, it is urgent to explore the combination regimen with complementary molecular mechanisms to reduce the side effects and enhance sensitivity of Doxorubicin. EGCG is a polyphenol in green tea with antitumor bioactivity which has been reported to show potential synergism with chemotherapy drugs in a few tumor types [6, 7]. Particularly, EGCG was found to have synergistic interaction with doxorubicin to kill the hepatoma Hep3B cells. Therefore, we hypothesized that EGCG and DOX also have synergistic inhibitory effects on osteosarcoma. Through the implementation of MTT experiment in which EGCG and DOX were used alone or in combination, the coefficient of drug interaction (CDI) was calculated and basically proved that they had synergistic effect on inhibiting the growth of osteosarcoma. The unknown molecular mechanism is another focus of this study.

DOX treatment could induce pro- survival autophagy in cells [26–28]. At the same time, EGCG promoted the growth inhibition effect of antitumor drugs through autophagy regulation via activation of pro-apoptosis or pro-survival autophagy in cancer cells [3, 29, 30]. Based on the synergistic effect of EGCG and DOX on osteosarcoma growth inhibition, in second part of this study, it was confirmed that EGCG inhibited DOX induced pro-survival autophagy which would be one of the important molecular mechanism of synergistic effect of EGCG and DOX on osteosarcoma. by detecting the expression of autophagy markers and immunofluorescence of LC3.

However, clarification of the targets of EGCG in this process was what attracts us to continue to explore. Our team was conducting exploratory functional studies of a series of LncRNAs in OS and fortunately, we found suspected targets of EGCG among them.

SOX2 overlapping transcript (SOX2OT) is a conserved lncRNA which encompasses sex determining region Y-box2 (SOX2) in the same strand [31]. SOX2OT have 8 transcript variants which expressions specifically in different human cancer cell lines [12]. In order to clarify differential expression of SOX2OT-spliced variants in OS tissue samples, 10 pairs of OS tissue samples and their relative adjacent tissues were collected from the second Xiangya hospital from Dec, 2015 to March, 2016. Unfortunately, RNA degradation was found in 4 pairs of tissues which could not be used in subsequent experiments. Since the expressions of variant 1, 4, 6, 7, 8 in tumor tissues are relatively common, and glioma cell line U87 could be used as a positive control of variant expression, we used RT-PCR method to detect variants in OS tissues. According to the results, SOX2OT variants 1, 4, 7 were found expressed in OS tissues. However, whether variant 1, 4, 7 are all meaningful for osteosarcoma tumorigenesis is still unknown. Next, we confirmed the expression of variants 1, 4, 7 in the corresponding tumor tissues and their adjacent tissues again and found that only SOX2OT variant 7 showed obvious over-expression in tumor tissues compared with their adjacent tissues while neither V 4 nor V 1 showed obvious tendency of expression in tumor or adjacent tissues. Based on the above, we locked SOX2OT V7 as the main object of our study in OS although it was not enough to arrive at convincing and statistically significant conclusions because only 6 pairs of OS tissue samples were tested. Fortunately, we found that V7 seems to be one of the important targets in the study of EGCG inhibiting autophagy induced by DOX and then the follow-up experiments basically confirmed EGCG inhibited DOX-induced autophagy by targeting SOX2OT V7 partially.

Cancer stem cells are considered to be closely related to drug resistance of tumors because of enhanced expression of ABC transporter proteins and activated autophagy [32, 33]. In view of the inhibition of the SOX2OT V7 expression in osteosarcoma cells of EGCG and the close relationship between SOX2OT and CSC pluripotent regulator SOX2, we put forward the hypothesis that EGCG could inhibit OSCs which would be another mechanism contributes to the Synergistic Interaction with Doxorubicin to osteosarcoma. EGCG could inhibit many kinds of cancer stem cells. But whether EGCG has an inhibitory effect on osteosarcoma stem cells has not been studied yet [34–36]. Through subsequent experiments, we have confirmed that EGCG can partially inhibit the self-renewal ability of OSC by targeting SOX2OT V7, and significantly enhance the inhibitory effect of DOX on OSC growth.

EGCG targeting lncRNA SOX2OT V7 inhibits pro-survival autophagy induced by chemotherapeutic drugs (such as DOX), thereby enhancing the chemosensitivity of osteosarcoma cells; At the same time, EGCG targeting SOX2OT V7 decreases stemness of osteosarcoma stem cells (including drug resistance, tumorigenic ability, self-renewal ability, etc.). Notch signaling pathway is an evolutionarily conserved signaling pathway that controls the fate of cells and maintenance of stemness [37]. EGCG could inhibit Notch signaling pathway [22–24, 38], Especially, it was found that Notch signaling pathway inactivation can effectively reduce cisplatin induced osteosarcoma stem cells production and reduce the resistance of osteosarcoma cells [39], which suggested that Notch signaling pathway play a role in OS drug resistance and stemness. In our research, by detecting downstream target genes of Notch signaling (Hes1 and Hey1), it was proved that V7 overexpression could further activated Notch signaling and EGCG treatment could inhibit the Notch signaling pathway in OSCs. To further understand the factors involved in this process, Notch receptors Notch1/2/3 and ligands such as Jagged1/2, Dll1/3/4 were invested by qRT-PCR. The results showed that in one hand there were no obvious expression alterations of Notch1 in OSCs derived from both U2OS and SaoS2 and of Notch 2 in OSCs derived from SaoS2 with or without V7 overexpression; in the other hand, Notch ligands Jagged 1, DLL1 and DLL4 showed no obvious expression alterations in OSCs derived from both U2OS and SaoS2 with V7 overexpression, besides, the expressions of Jagged2 were even not significantly changed in any groups including in OSCs compared with parental group. According to the results of this study, in all ligands and receptors of the Notch signaling, only Notch 3 and DLL 3 met the expectations of this research. So, we conclude the OSC inhibitory effect of EGCG targeting SOX2OT V7 is partly achieved by restrain of Notch3 / DLL3 signaling.

## Conclusions

EGCG targeting LncRNA SOX2OT variant7 via Notch3/DLL3 plays a role in autophagy inhibition and stemness reduction which contributes to the synergistic interaction with Doxorubicin to osteosarcoma.

## Abbreviations

CDI: The coefficient of drug interaction
CSCs: Cancer stem cells
Dox: Doxorubicin
EGCG: Epigallocatechin gallate
LncRNA: Long non-coding RNAs
OS: Osteosarcoma
OSCs: Osteosarcoma stem cells
SOX2OT: Human SOX2 Over lapping transcript
